# Construct validity of the interview Time Trade-Off and computer Time Trade-Off in patients with rheumatoid arthritis: A cross-sectional observational pilot study

**DOI:** 10.1186/1471-2474-13-112

**Published:** 2012-06-25

**Authors:** Laurien Buitinga, Louise MA Braakman-Jansen, Erik Taal, Mart AFJ van de Laar

**Affiliations:** 1 Department of Psychology, Health and Technology, Institute for Innovation and Governance Studies, University of Twente, Enschede, The Netherlands; 2 Department of Rheumatology and Clinical Immunology, Medisch Spectrum Twente, Enschede, The Netherlands

## Abstract

**Background:**

The Time Trade-Off (TTO) is a widely used instrument for valuing preference-based health-related quality of life (HRQoL). The TTO reveals preferences for own current health (‘utilities’) on a scale anchored between death (0) and perfect health (1). Limited information on the external validity of the TTO is available. Aim of this pilot study was to examine the construct validity of both an interview TTO and a computer-based TTO in patients with rheumatoid arthritis (RA).

**Methods:**

Thirty patients visiting the outpatient rheumatology clinic participated. Construct validity was assessed by measuring convergent and discriminative validity. Convergent validity was assessed by calculating Spearman’s correlations between the utilities obtained from the TTOs and pain, general health (rating scales), health-related quality of life (SF-36 and SF-6D) and functional status (HAQ-DI). Discriminative power of both TTO measures was determined by comparing median utilities between worse and better health outcomes.

**Results:**

Correlations of both TTO measures with HRQoL, general health, pain and functional status were poor (absolute values ranging from .05 to .26). Both TTOs appeared to have no discriminative value among groups of RA patients who had a worse or better health status defined by six health outcome measures. About one-third of respondents were zero-traders on each of the TTO measures. After excluding zero-traders from analysis, the correlations improved considerably.

**Conclusions:**

Both the interview TTO and computer TTO showed poor construct validity in RA patients when using measures of HRQol, general health, pain and functional status as reference measures. Possibly, the validity of the TTO improves when using an anchor that is more realistic to RA patients than the anchor ‘death’.

## Background

The Time Trade-Off (TTO) [1] is an instrument developed to assess effects of treatments in cost-utility analyses (CUAs) by measuring changes in health-related quality of life (HRQoL) directly by patients. The TTO reveals preferences for own current health (‘utilities’) on a scale anchored between death (0) and perfect health (1) by asking people how many life years they are willing to give up to become perfectly healthy. It is assumed that the more life years people are willing to trade off, the worse their health state is. The purpose of this measure is to capture the desirability of patients’ own health state reflecting their health-related quality of life (HRQoL).

Traditionally, the TTO is administered by interview. The TTO can also be administered by questionnaire or computer. Furthermore, different methodological approaches to the TTO are used [2]. This makes comparison between studies difficult. Differences in TTO procedures seem to influence utilities. For example, it has been found that utility scores are heavily influenced by the method of elicitation (ping-pong, titration) [3]. Furthermore, the mode of administration (interview/computer/questionnaire) or the way the TTO question is formulated can influence utilities. Besides, the size of time frame that is used (e.g. fixed time period, life expectancy) has a great impact, since utilities are calculated as the proportion of the remaining lifetime sacrificed [2].

Few studies have examined psychometric properties of the interview TTO in rheumatoid arthritis (RA). The studies that reported on the construct validity, showed poor to moderate correlations between TTO and measures of HRQoL, functional status, disease activity and pain [4-6]. It was found that the TTO was only able to discriminate between worse or better disease-specific HRQoL using the RAQoL [4,5], between worse or better outcomes on the dimensions ‘symptom’ and ‘role’ of the disease-specific AIMS-2 [5] and between worse or better mental health using the RAND-36 mental component summary scale [4]. Tijhuis et al. showed that the TTO was able to discriminate between worse or better pain, worse or better disease activity and worse or better functional status [4]. In contrast, Bejia et al. showed that the TTO was not able to discriminate between worse and better pain or worse and better disease activity [5].

Computer-based utility elicitation procedures to administer the TTO have been developed, for example iMPACT3 [7] and U-Titer [8]. Studies in a range of conditions have used such computer-based programmes to administer a TTO using different procedures [7,9].

In this study, we report on preliminary results with respect to the construct validity of the TTO assessed in patients with RA using an interview TTO as well as a computer TTO, and using a standardised procedure for both TTOs. The first aim of this study was to examine convergent validity of the interview and computer TTO separately by correlating TTO utilities of both TTO measures with other patient-reported outcomes (PROs) in patients with RA. The second aim was to examine whether the interview and computer TTO were able to discriminate between worse and better patient-reported health outcomes.

## Methods

### Patients and study design

Thirty consecutive outpatients (aged 18–85) of our rheumatology clinic who were diagnosed with RA participated. People who did not understand the Dutch language were excluded.

All participants completed the TTO twice with an interval of 14 days. Randomly the first TTO was either interview or computer-based, consequently followed by the other at the next assessment. Measures of pain, general health, health-related quality of life and functional status were administered at the first TTO assessment. Informed consent was obtained from all participants. According to legislation in the Netherlands (WMO), no approval of the ethical review board was indicated.

### Measures

#### TTO interview

The Time Trade-Off question used in this study was formulated as follows:

“Imagine that a new treatment became available which helped you to recover fully. A side-effect of this treatment, however, is that you will die sooner. Would you opt for this treatment?” A graphical aid was used to make the question more clearly. When participants asked about the definition of being perfectly healthy, they were told to imagine being in perfect health without any disease or health-related complaints.

A life time perspective was adopted. Life expectancy calculations of the Dutch Central Bureau of Statistics [10] were used. The remaining life expectancy was calculated by extracting the age of the participant from his or her expected age of dying according to the CBS. The bisection method was applied to reach the point at which participants did not prefer one of the two options: staying in their health state for the rest of their lives or being perfectly healthy for a shorter life time. Therefore, the trade-off started with setting the shorter life in perfect health on half of the remaining life expectancy. For example, a person with a remaining life expectancy of 20 years was first asked about his or her willingness to trade off 10 life years. If the person accepted the trade, a remaining life expectancy of five years in perfect health was presented. If the person did not accept the trade, a remaining life expectancy of 15 years was presented. This process continued until the patient was indifferent between his or her own current health state according to his or her life expectancy and a shorter life in perfect health. Then, the TTO score was calculated by the formula: 1-(number of life years given up/remaining expected life years).

#### Computer TTO

**Figure 1:**
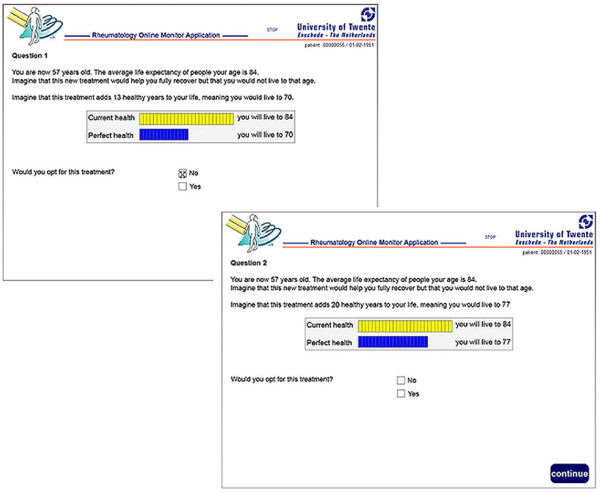
Two screen shots of the computer TTO*. Here, a person with a life expectancy of 27 years was asked about his or her willingness to trade off 13 life years. The person did not accept the 13 years in perfect health, so six years were added in the second question. *for this publication the screen shots were translated from Dutch to English.

Utilities were obtained by means of touch screens. Graphical presentations supported the TTO question (Figure 1). The computer TTO and the interview TTO were formatted equally, using similar formulations and graphical presentations. The bisection method was used to find the indifference point. During the computer assessment of the TTO, the researcher (LB) was present to start up the computer. The respondents completed the TTO independently. The researcher observed the patients during the assessment. Our previous study [11] demonstrated that the test retest-reliability of this computer TTO was good (ICC = 0.88) and comparable with the test reliability of the interview TTO [4,5].

#### NRS pain and general health

Current severity of pain and current general health were both measured by a numerical rating scale (NRS), ranging from 0 (best) to 10 (worst).

#### SF-36

Physical and mental health were measured by calculating the physical and mental component summary scores (PCS and MCS) of the SF-36 version 2 [12], a generic descriptive instrument for measuring health-related quality life on eight dimensions (mental functioning, physical functioning, bodily pain, vitality, role limitations due to physical problems, role limitations due to emotional problems, social functioning and general health). The scores range from 0 to 100, whereby a higher score indicates a better health.

#### SF-6D

From the SF-36, SF-6D utility scores were derived, reflecting health state valuations of the general public [13]. The utility scores range from 0 to 1, whereby a higher score indicates a better HRQoL.

#### HAQ-DI

The level of functional disability was assessed by the Health Assessment Questionnaire Disability Index (HAQ-DI) [14], a self-report measure consisting of eight categories (dressing and grooming, arising, eating, walking, hygiene, reach, grip and common daily activities). The HAQ score ranges from 0 to 3, whereby a higher score indicates a worse functional status.

#### Statistical analysis

To examine the presence of an order effect between participants who started with the interview TTO or with the computer TTO, a Mann–Whitney *U*-test was performed.

Construct validity was assessed by measuring convergent and discriminative validity. Convergent validity of the interview and computer version was assessed by calculating Spearman’s correlations between each of the TTOs with the NRS for pain and general health, SF-36, SF-6D and HAQ-DI. Moderate correlations (0.40-0.59) are expected: all measures (except for the SF-6D) are descriptive, and most instruments only capture one or some aspects of the construct quality of life. The SF-6D yields utilities, but these are derived from a general public. A sample of 29 participants is required to demonstrate a significant moderate Spearman’s correlation of 0.50 with an alpha of 0.05 (one-tailed) and a power (1-β) of 0.80. Discriminative power of the interview TTO and computer TTO was determined by comparing median utilities between worse and better pain, general health, HRQoL and functional status. Therefore, the outcome measures were dichotomised by the median score. A worse health outcome was defined by the ≤ median value of the outcome measure. A better health outcome was defined by the > median value of the outcome measure. Because of a difference in scaling of the NRS (Pain and General Health) and HAQ-DI, a worse health outcome on these instruments was defined by the > median value of the outcome measure. A better health outcome was defined by the ≤ median value of the outcome measure. The Mann–Whitney *U*-test was used to test significance. Data were analysed using SPSS version 16.0.

## Results

### Subjects

All 30 patients who participated completed both TTO measures. Demographic, clinical and psychosocial characteristics and utility scores for both TTO methods of the 30 patients are shown in Table 1. Median TTO utility scores were 0.87 (interview TTO) and 0.85 (computer TTO). Ten participants did not want to trade any life year for perfect health on the interview TTO; eight participants did not want to trade any life year for perfect health on the computer TTO (zero-traders). Zero-traders did not have a significantly different self-reported health than traders (data not shown). Six zero-traders on the computer TTO were also zero-traders on the interview TTO. Two zero-traders on the computer TTO were not zero-traders on the interview TTO, and two zero-traders on the interview TTO were not zero-traders on the computer TTO.

**Table 1:** Demographic, clinical and psychosocial characteristics

	**N (30)**
**Age (mean years ± SD)**	58 ± 13
**Gender (%)**	
Men	27
Women	73
**Disease duration (median years (IQR))**	10 (19–3)
**Marital status (%)**	
Single	17
Married/Living together	83
**Educational level (%)**	
Low	50
Moderate	30
High	20
**Work status (%)**	
Paid work	40
Housekeeping	10
retired/unemployed/disabled	50
**Utilities (Median (IQR))**	
**Interview TTO**	0.87 (0.80-0.87)
**Computer TTO**	0.85 (0.58-0.85)
**Pain (numerical rating scale) (median (IQR))**	4 (2–6)
**General Health (numerical rating scale) (median (IQR))**	5 (3–6)
**Descriptive health-related quality of life (SF36) (median (IQR))**	
**Physical**	40.05 (34.22-46.73)
**Mental**	40.99 (35.68-43.95)
**Preference-based health related quality of life (SF-6D) (median (IQR))**	0.63 (0.59-0.75)
**Functional status (HAQ-DI) (median (IQR))**	0.88 (0.34-1.38)

### Test order

For neither interview TTO utility scores nor computer TTO utility scores an effect of test order was found (P = 0.37 and P = 0.73 respectively). So, no significant differences in utility scores existed between patients who started with the interview TTO or computer TTO.

### Construct validity: Convergent and discriminative validity

Correlations between utilities and scores on rating scales for pain and general health, SF-36, SF-6D and HAQ-DI are shown in Table 2. In the entire group of RA patients, poor correlations were found between either both TTOs and the NRS measuring pain and general health, physical and mental component summaries of the SF-36, the SF-6D and the HAQ-DI. After exclusion of zero-traders, the correlations were moderate and significant between either the interview TTO and the NRS measuring pain (r = −0.38) and general health (r = −0.42), the physical component summary of the SF-36 (r = 0.45), and the SF-6D (r = 0.45). In addition, moderate and significant correlations were found between either the computer TTO and the NRS pain (r = −0.47) and the HAQ-DI (r = −0.38) (Table 2). All other correlations remained non-significant after exclusion of zero-traders. Discriminative properties of both TTOs are shown in Table 3. Both TTOs proved to have no discriminative value between worse and better health outcomes for all six outcome measures. Performing these analyses without the zero-traders resulted in larger and significant median differences in interview TTO scores between worse and better outcomes for the physical component summary of the SF-36 (difference in mean rank = 5.50), the NRS measuring pain (difference in mean rank = 5.83) and the SF-6D (difference in mean rank = 4.85). In addition, larger and significant median differences in computer TTO scores between worse and better outcomes were found for the NRS measuring pain (difference in mean rank = 6.30). No significant median differences in TTO scores between worse and better health outcomes were found for the other outcome measures.

**Table 2:** Spearman’s correlations (95% confidence intervals) of interview TTO or computer TTO utilities with health outcome measures for the total sample and for the traders only

	**Interview TTO**	**Interview TTO traders only**	**Computer TTO**	**Computer TTO traders only**
	**(N = 30)**	**(N = 20)**	**(N = 30)**	**(N = 22)**
**Pain (NRS)**^≈^	−0.10 (−0.45, 0.27)	−0.38 (−0.70, 0.08)*	−0.26 (−0.57, 0.11)	−0.47 (−0.74, -0.05)*
**General Health (NRS)**^≈^	−0.08 (−0.43, 0.29)	−0.42 (−0.73, 0.03)*	−0.05 (−0.40, 0.32)	−0.13 (−0.53, 0.30)
**Descriptive HRQoL (SF-36)**				
**PCS**	0.16 (−0.22, 0.49)	0.45 (0.01, 0.74)*	0.22 (−0.15, 0.54)	0.35 (−0.08, 0.67)
**MCS**	0.24 (−0.14, 0.55)	0.32 (−0.14, 0.67)	0.22 (−0.15, 0.54)	0.24 (−0.20, 0.60)
**Preference-based HRQoL (SF-6D)**	0.18 (−0.21, 0.50)	0.45 (−0.01, 0.73)*	0.11 (−0.27, 0.45)	0.20 (−0.24, 0.57)
**Functional status (HAQ-DI)**^≈^	−0.07 (−0.42, 0.29)	−0.20 (−0.59, 0.27)	−0.21 (−0.53, 0.17)	−0.38 (−0.69, 0.05)*

NRS = numerical rating scale; HRQoL = health-related quality of life; PCS = Physical Component Summary; MCS = Mental Component Summary.

^≈^ Negative correlations were caused by a difference in scaling between the TTO and three health outcomes: a higher TTO score means a better HRQoL, whereas a higher score on these health outcomes means a worse health outcome.

*Significant correlations (P < 0.05).

**Table 3:** **Discrimination of the interview and computer TTO between worse and better health outcomes**^**a d**^

	**Interview TTO utilities (N = 30)**			**Computer TTO utilities (N = 30)**		
	***Worse health outcomes***^**b c**^	***Better health outcomes***	***P***	***Worse health outcomes***^**b c**^	***Better health outcomes***	***P***
**Pain (NRS)**^e^	0.87 (0.81-0.97)	0.87 (0.57-1.00)	0.48	0.60 (0.50-1.00)	0.85 (0.74-0.97)	0.15
**General Health (NRS)**	0.87 (0.79-0.99)	0.93 (0.77-1.00)	0.31	0.87 (0.58-1.00)	0.82 (0.57-0.98)	0.23
**SF-36 PCS**	0.86 (0.67-1.00)	0.88 (0.80 -1.00)	0.24	0.77 (0.53-1.00)	0.85 (0.71-1.00)	0.26
**PCS**	0.87 (0.63-1.00)	0.87 (0.80 -1.00)	0.45	0.84 (0.50-1.00)	0.85 (0.60-1.00)	0.28
**SF-6D**	0.87 (0.79-1.00)	0.88 (0.78-0.96)	0.36	0.86 (0.58-1.00)	0.81 (0.58-0.95)	0.29
**HAQ-DI**	0.91 (0.82-1.00)	0.85 (0.70-0.99)	0.48	0.86 (0.52-1.00)	0.85 (0.59-0.92)	0.37

^a^ Utilities are expressed in median scores (interquartile range); ^b^ For NRS (Pain), NRS (General Health) and HAQ-DI: a worse health outcome was defined by the > median value of each outcome measure; ^c^ For the SF-36 and SF-6D: a worse health outcome was defined by the ≤ median value of each outcome measure; ^d^ Median values for the NRS (Pain), 4; for the NRS (General health), 5; for the SF-36 PCS, 40.05, MCS, 40.99; for the SF-6D, 0.63; for the HAQ-DI, 0.88; ^e^ NRS = numerical rating scale.

## Discussion

This pilot study showed that the construct validity of both the interview TTO and computer TTO was poor in patients with RA when using measures of HRQol, general health, pain and functional status as reference measures. After exclusion of zero-traders from analysis, the results improved. This finding was expected, because zero-traders did not have a significantly different health status compared with traders. Indications of the poor convergent validity of the TTO were also found in other studies in RA and studies in other diseases [4-6,9,15-17]. In most of these studies it was unclear how many participants were zero-traders and whether they were in- or excluded. One study reported similar results when in- or excluding zero-traders from analysis [15]. In our study, we did not find the TTO to be discriminative for any of the health outcome measures used. Other studies found evidence for and against its discriminative ability [4,5,9,16]. Contradicting findings were found for pain and disease activity scores in patients with RA [4,5] and for functional status scores in patients with cardiovascular disease [9,16].

All these studies were found to have differences in the TTO procedure applied. This might explain the contradicting results regarding the discriminative ability of the TTO. Beside the mode of administration, studies differed in the time frame used (remaining life expectancy [4,5,16-18], time frame dependent on age group [6] or not mentioned [15]). Furthermore, some studies described the way in which people had to think about current health [16,17] and/or about the anchors perfect health [4,5,16] and death [16], whereas other studies did not [6,9,18]. One study used a symptom-free anchor (‘no angina’) instead of ‘perfect health’ [9]. In many studies it was stated that a visual aid was used, although no further information was given about its representation [4-6]. Besides, many studies did not report the precise method of elicitation (e.g. ping-pong) [4-6,9,18].

In our study, the TTO procedure applied was precisely described, facilitating the comparison with other studies. Strengths of this study were the fact that we used two different TTO assessments and that we used a broad set of PROs in a homogeneous population consisting of RA patients. A limitation of this study was the use of a small convenience sample.

There are several explanations possible for the results of our study, irrespective of the TTO procedure used. First, the low correlation with the SF-6D, another preference-based instrument, can be partly explained by the difference in perspective used to obtain utilities. SF-6D utilities are derived from the general public, so these scores represent a societal perspective. TTO scores were directly calculated from the patients’ preferences, representing a patient perspective. Secondly, except for the SF-36 and SF-6D, the comparators used in this study only measure one aspect (e.g. functional status) of the construct quality of life. Furthermore, except for the SF-6D, the comparators are descriptive which implies that valuations of health states are not assessed. With these measures patients are asked about their levels of impaired health or pain, whereas personal preferences toward their health state remain unrevealed. It is possible that people with the same health state report different utilities if they have different ‘aspirations’ [18]. Nease et al. illustrate this by the example that inability to walk ‘more than a city block’ does not have to be a limitation if someone does not desire to be active [18]. Therefore, it would be worthwhile to examine in future studies whether it is better to validate the TTO against individualized measures of personal preferences, such as the SEIQOL [19,20] or MACTAR [21]. Thirdly, it has been found that preferences are prone to biases inherently to the nature of the TTO, such as loss aversion. Loss aversion can be observed when a choice has to be made between ‘remaining the status quo’ (remaining in the current health state) and ‘accepting an alternative to it’ (trading off life years for perfect health). In that case people will evaluate the advantages and disadvantages of the alternative in terms of losses and gains [22]. The TTO asks people about their willingness to trade off life years (a loss) for optimal health (a gain) [23]. Because ‘losses loom larger than gains’ [22], people become reluctant to give up life years. This will result in higher utilities, as supported by findings of Van Osch et al. [24]. Furthermore, TTO utilities might be influenced by other factors that are unrelated to current health [15], such as family-related aspects, for example having children [2] or seeing grandchildren grow up [17]. Finally, the nature of the disease can influence utilities. Asking patients to trade off life years may feel unrealistic, because patients with RA do not perceive their disease as life-threatening [6]. Therefore, people may be less willing or not willing at all to trade off life years. Our results are indicative of this: irrespective of health, a relatively large number of participants were not willing to trade any life year for perfect health. For chronic illnesses such as RA there may be more realistic health-related anchors, for example ‘becoming dependent on others’ and ‘having increased physical limitations’, which were reported by RA patients to worry them [25,26]. It could be examined whether the validity of the TTO improves when changing the trade-off about dying earlier in other more realistic (health-related) trade-offs. The use of a ‘chained’ TTO procedure could also improve the validity of the TTO. In a chained procedure, the health state of interest is not directly compared with death but indirectly with the aid of an intermediate anchor health state [27-29]. A limitation is that a chained procedure is more complex, because it adds an additional step to the valuation process, possibly leading to extra noise [28]. Limited research has been performed on the chained TTO and has been mainly applied in temporary health states [28-30]. For chronic health states it has been shown that chained TTOs are systematically biased upwards (when the worst endpoint was varied) or downwards (when the best endpoint was varied), but that it is possible to correct for these biases [31]. However, the respondents were not patients, but healthy people and women at high risk for breast cancer. Research in chronically ill patients examining the validity of the chained TTO for chronic states is lacking.

## Conclusions

In conclusion, both the standardised interview TTO and standardised computer TTO showed similar poor results regarding construct validity when using measures of HRQoL, general health, pain and functional status as reference measures. Possibly, the validity of the TTO can be improved by replacing the anchor ‘death’ by an anchor that is more realistic to RA patients. Future studies in which direct patient reported utilities are derived, could start with the development of a TTO instrument using realistic anchors for RA patients. This instrument could be validated against individualized measures of personal preferences, such as the SEIQOL or MACTAR instrument.

## Competing interests

The authors declare that they have no competing interests.

## Authors’ contributions

LB was the primary researcher, responsible for collection of data by administering the instruments, data analysis and writing the manuscript. LMABJ, ET and MAFJL were closely involved in designing the study, interpreting the results and revising the manuscript. All authors have read and approved the final manuscript.

## Pre-publication history

The pre-publication history for this paper can be accessed here:

http://www.biomedcentral.com/1471-2474/13/112/prepub
